# Multi-Year Multi-Indication Agreements for Supporting Patient Access to Oncology Medicines with Multiple Indications: An Experimental Approach or Here to Stay?

**DOI:** 10.3390/jmahp13010002

**Published:** 2025-01-24

**Authors:** Hannah Armstrong, Angelina Petrova, Tim Wilsdon, Henriette Homoki, Alexander Roediger

**Affiliations:** 1 Charles River Associates, London EC2M 7EA, UK; 2 MSD International Business GmbH, 6010 Kriens, Switzerland

**Keywords:** multi-indication, multi-year, access agreements, oncology, immuno-oncology

## Abstract

Over the past decade, an increasing number of oncology medicines with indications for multiple cancer types have been delivering benefits to patients. To ensure these products reach patients, pricing and reimbursement systems have had to adapt to address the value assessment, time-to-access, affordability, and budget uncertainty challenges this creates. Multi-year multi-indication (MYMI) agreements are made between payers and manufacturers and aim to ensure that patients have access to effective treatments for multiple conditions over time; this includes future indications of the treatment. MYMI agreements were first introduced as a solution in several European countries in 2017, offering a range of potential benefits. MYMI agreements have since demonstrated evidence of success in mitigating many of the challenges associated with assessing and reimbursing multi-indication products, time-to-patient access, and budget impact. The purpose of this article is to discuss the recent progress made with MYMI implementation across countries and provide a view on whether it is delivering for patients, healthcare systems, and innovators. We find that MYMI is not a one-size-fits-all solution but a model that needs to be adapted to the unique needs and characteristics of different healthcare systems. The intended benefits of MYMI to patients (speed and breadth of access to new indications) appear to have been realised in practice in some countries but not all. However, the administrative burden associated with MYMI implementation in some countries risks jeopardising the intended efficiency benefits. Payers and policymakers can also benefit from improved budget predictability and sustainability.

## 1. Introduction

Over the past decade, an increasing number of innovative medicines have obtained marketing authorisation in multiple indications. Predominantly, this trend is noticeable in the oncology space driven by research in immuno-oncology, specifically research in immune checkpoint inhibitors such as PD-1 and PD-L1 inhibitors, where drugs have demonstrated efficacy across a large number of indications. As described in depth by Lawlor et al. (2021) [1], this creates several challenges for patients, payers, and the manufacturers of the medicines. The most significant of these is that patients in different countries do not always have access to valuable indications, or access is significantly delayed. Multi-indication products are resource-intensive for Health Technology Assessment (HTA) bodies to assess, and the assessment of a product with multiple indications is further complicated by the likelihood that the ’value’ of this product to patients and the healthcare system differs across indications. Additionally, multi-indication products introduce budget uncertainties and difficulties for payers, as it is challenging to predict the number of future indications.

Following the review by Lawlor et al. published in 2021, there has been continued growth in the number of indications in which PD-(L)1s have demonstrated clinical value. As of May 2022, there were over 5500 clinical trials that included a PD-(L)1-targeting agent [2].

Since the emergence of multi-indication products, pharmaceutical companies in tandem with healthcare systems have been developing ways to minimise the challenges associated with the assessment and reimbursement of such medicines. Specifically, this has included discussions of alternative access arrangements that would support patient access by streamlining the HTA processes. In 2021, Lawlor et al. concluded that multi-year multi-indication (MYMI) access agreements, made between payers and manufacturers for products and created to go across multiple indications and years, provide a more pragmatic approach to HTA for medicines with multiple indications to ensure both fast and broad patient access [1]. MYMI agreements are intended to complement the HTA process rather than substitute it. While the clinical and economic value of different patient populations and treatment lines is evaluated through the HTA process, MYMI agreements focus on streamlining access and improving budget predictability.

Now, seven years after the first MYMI agreement was negotiated, we have looked at the impact of these agreements across countries and considered the extent to which they have succeeded in balancing patient access and affordability of multi-indication products. The majority of MYMI agreements remain confidential; therefore, in this paper, we focus on four European countries where the existence of MYMI agreements is supported by evidence available in the public domain.

The remainder of the paper is structured as follows: In Section 2, we describe how the MYMI concept has been implemented in practice in four European countries; in Section 3, we set out opinions on the advantages and disadvantages that MYMI agreements have provided; and in Section 4, we offer conclusions on the role that MYMI agreements have played and could continue to play in addressing challenges associated with access to multi-indication medicines in oncology.

## 2. Experiences with MYMI Agreements

### 2.1. Belgium

As has been documented in the previous study, the initial development of MYMI agreements in Belgium occurred under a broader framework agreed between the government and industry, known as the Pact of the Future [1,3]. For PD-(L)1s, this led to the earliest documented adoption of an MYMI agreement in Europe, in 2017, the terms of which were that all PD-(L)1s approved for reimbursement by the Minister of Social Affairs would have new indications reimbursed within one month of approval by the European Medicines Agency (EMA); this has been referred to as EMA+1 [4]. This agreement would, theoretically, allow faster access to treatments without affecting the reimbursement price, as the prices would be negotiated by the manufacturer. Similarly structured innovative access agreements have continued to be used for PD-(L)1s for the last seven years in Belgium, and the first agreement was recently renewed again.

Recent evidence suggests that the EMA+1 agreement can enable faster and wider patient access to new PD-(L)1 indications. In Belgium, the official time for the Minister of Social Affairs to make a decision regarding the reimbursement of a new indication is 180 days, in line with EU Directive 89/105 (the Transparency Directive) [5], although, in practice, this process can take, on average, 594 days for oncology medicines as a result of clock stops and delays due to the value assessment process [6]. Under an MYMI agreement, due to the involvement of EMA+1, access for patients is granted within 30 days of EMA approval. The MYMI agreement for PD-(L)1s could therefore be enabling patients to obtain access to new indications over 550 days earlier than would otherwise be possible.

### 2.2. The Netherlands

The previous paper reported that in the Netherlands, MYMI agreements evolved from an initial policy precedent established in 2016 that enabled manufacturers to negotiate multi-year contracts for products with a single indication [1]. Following this, true MYMI agreements could then be established on a per-product basis. Although the terms of the MYMI contracts in place in the Netherlands were originally and remain confidential, it is understood that agreements largely take the form of multi-year price–volume agreements; any additional EMA-approved indication can be added to the agreement. Price–volume agreements link the price of a product with the volume of sales; we believe that in some confidential agreements the price is linked to the combined volume of sales across all indications [7]. While single-indication price–volume agreements seem to be a relatively common way for the National Health Care Institute (ZIN) to control expenditure on medicines with a potentially high budget impact, the multi-indication structure is believed to be rarer [1]. This satisfies the criteria of an MYMI agreement, as it spans both time and treatment indications. ZIN does not carry out a full HTA evaluation of each new indication, but instead the scientific committee (CieBOM) of the Dutch Association for Medical Oncology enables reimbursement by issuing a clinically based recommendation, which should be subsequently adapted for reimbursement via the healthcare insurers [1]. However, more recently, positive CieBOM advice has not always led to reimbursement (as highlighted in other studies), and assessments by healthcare insurers for multiple indications have been delayed [6,8].

We believe that the benefits of the style of MYMI agreement used in the Netherlands are the efficiencies created for the HTA body and ZIN, and thus the potential accelerated reimbursement of new indications. With the reimbursement of new oncology medicines and indications taking an average of 320 days in the Netherlands [6], and capacity constraints being reported at HTA agencies across Europe [9], bypassing a full HTA evaluation of each new indication could theoretically alleviate the burden on the HTA body and accelerate patient access. We therefore believe that, in practice, avoidance of a full HTA will result in a slightly improved speed of access. Additionally, the recently issued requirement to await the completion of the scientific appraisal process by CieBOM generates some delays.

Although it is understood that, under the terms of the MYMI agreement, a full retrospective HTA evaluation can be conducted by ZIN, there is no evidence that this has been applied so far in practice, except in the case of combination products which are not under an MYMI agreement. Workload efficiency for payers, therefore, remains a key benefit; however, the wider potential benefits of MYMI agreements are not being fully realised in the Netherlands.

### 2.3. Greece

In Greece, once a product or indication has been positively assessed by the Committee for the Evaluation and Reimbursement of Medicines for Human Use, its reimbursement price will be negotiated with the Pharmaceutical Price Negotiation Committee [10]. Since 2018, an Electronic Prior Authorisation System (EPAS) has become a standard practice for all products reimbursed for serious diseases and has resulted in faster patient access to innovation—as fast as approximately six months after EMA approval for new products and two months for new indications, as the latter products have been already included in the reimbursement list [11]. Although this system enables faster access for new medicines and indications, it does not guarantee equitable treatment for all patients, as it is up to the physician to initiate the process. In addition, it can create affordability concerns for the Ministry of Health (MoH), which then need to be mitigated through cost-shifting mechanisms, for example through clawbacks [12].

Rising clawbacks in recent years have created an impetus to find alternative solutions. Between 2012 and 2019, the combined value of pharmaceutical clawbacks and rebates in Greece rose by approximately 420% [13]. Although clawbacks can support healthcare budget management, their uncontrolled growth creates financial unpredictability for payers. Following the COVID-19 pandemic, there was further pressure from the European Parliament (via the EU Recovery and Resilience Fund) requiring clawback levels in Greece to fall by 2025 [14]. For PD-(L)1s, this drove the establishment of closed product budget caps—based on the agreed volume and price discount—separate from the total pharmaceutical budget, under which spending on new indications for each product over multiple years can be better managed. Product-specific budget caps (and thus product-specific clawbacks) may enable greater financial certainty for the MoH and for innovators in the face of unsustainable rising clawback rates. The MYMI agreement can therefore decrease total market clawback and thus contribute to the achievement of the respective target of EU’s Recovery and Resilience Fund.

Officially, the standard HTA and price negotiation process along with the MoH decision for reimbursement should be completed within 180 days, in line with the EU Transparency Directive [5]. However, empirical data from EFPIA’s W.A.I.T. indicator reveal that the average time to official reimbursement of a new product or indication in Greece is 568 days [6]. Theoretically, the MYMI agreement could help resolve some of the aforementioned delays. Regardless, we believe that the introduction of MYMI agreements has been beneficial for payers due to the budget predictability cultivated by budget caps.

### 2.4. Switzerland

For medicines with multiple indications, Switzerland has an indication-based pricing system (IBP), which has been applied in practice to many oncology medicines. IBP captures the value of a drug across various indications and establishes net prices based on the indication the product is used for; however, in many cases the negotiated net discounts are not confidential [15]. Further, implementation of IBP is challenging due to the lack of established systems to track different uses of the same medicine across its indications [15]. To address issues with delays and concerns around price transparency for innovative medicines, the Federal Office of Public Health started to allow for confidential pricing models. In 2020, the Ministry of the Interior proposed a new law (“Modèles de Prix Pour Médicaments”), to consolidate a formal basis for such pricing models [15]. The proposed law change still is in the parliamentary approval process; the National Council’s Committee on Social Security and Public Health discussed this proposal in June 2024, but they have not yet reached a consensus [16].

To track volume of use of medicines across indications, in 2021, administrative indications codes were established. This enabled the novel adoption of multi-year weighted pricing models for PD-(L)1s; a single price (confidential or transparent) can be established based on the relative volume of use across a product’s indications. The price can be renegotiated after a fixed multi-year period to account for the impact of new indications on the weighted price. In the latest models, it is possible to add new indications throughout the period of the MYMI, triggering a price re-evaluation. Overall, these confidential weighted pricing models have the potential to accelerate reimbursement timing, thanks to the confidentiality of the individual indication prices, and under certain circumstances can remove the disincentive manufacturers often face to launch larger, lower-priced indications in Switzerland [15].

## 3. Lessons from the Implementation of MYMI Agreements

### 3.1. How Has the Use of MYMI Agreements Evolved over Time?

The first MYMI agreements were signed for PD-(L)1s in 2017. In the seven years since their initial negotiation, these agreements have all been renegotiated due to their apparent practical and mutually beneficial nature for enabling patient access across multiple indications and years.

MYMI agreements can support economically challenging environments. Initially their application was limited predominantly to cost-effective markets such as Belgium and the Netherlands. However, because MYMI agreements can address budget predictability concerns, they are also attractive to markets with economic constraints and a stronger budget impact focus, such as Greece.

MYMI agreements need to be tailored to the country’s pricing and reimbursement system. Looking across all countries that have adopted MYMI agreements or use alternative pathways to handle challenges related to multi-indication products, it is clear there is no one-size-fits-all approach. Although a price–volume agreement may be the predominant model because of its relative simplicity, for MYMI to work for payers and for innovators it appears that it needs to be tailored to meet the primary objectives and to address the specific challenges and market access pathways in that country, including time to patient access, reduction of the administrative burden, and/or budget impact uncertainty. The need for a tailored approach has resulted in each country adopting additional nuanced characteristics on top of the ‘traditional’ core MYMI model in order to make this work in its healthcare system, such as HTA exemptions or HTA alternatives, budget caps, and clawback mechanisms.

Interestingly, we have not observed any strong role for international lesson-sharing amongst countries to align on the most optimal approach based on shared experiences. The literature on MYMI agreements remains very scarce. Given the absence of published reviews, we can infer that newer MYMI agreements appear to have been developed by payers without a systematic assessment of approaches applied in other countries and evidence of their impact.

### 3.2. Is There Evidence That MYMI Agreements Have Delivered Benefits for Patients, the Healthcare System, and Innovators?

Based on the available evidence, for patients, speed and breadth of access to new indications remain the key benefits of MYMI agreements. While speed of access was a primary benefit of earlier agreements, such as those highlighted in Belgium and the earlier ones in the Netherlands (delays have been recorded in recent years), MYMI agreements have not led to dramatically decreased wait times in the other markets. Often this is because MYMI is not used as a replacement for HTA. The Belgian EMA+1 structure with retrospective value assessment remains the best practice in terms of accelerating patient access to new indications. In some cases, the benefit is not speed but completeness of access to all new indications and setting of fixed prices during the contracted period. Patients can typically access more PD-(L)1 indications when an MYMI agreement is in place than when standard pricing and reimbursement pathways are followed, as MYMI removes many access barriers between EMA approval and reimbursement. A potential example of this is Keytruda and its assessment in Belgium. Keytruda is available in 22 indications in Belgium (out of the 23 that have been approved by EMA as of December 2023), and without the presence of an MYMI agreement it would have to go through an equivalent number of HTA reviews, delaying access and the potential availability of indications in the case of a negative decision.

The structures of MYMI agreements that have been adopted show that payers and policymakers also benefit from improved budget predictability and sustainability. For the innovator, faster patient access to more indications than would otherwise be attainable and greater financial predictability are benefits that innovators share with patients and payers.

The benefits of reduced assessments and reduced use of resources are not as significant as originally hypothesised. Over time, some markets, such as the Netherlands, have adapted the MYMI structure so there is still a value assessment by indication, as would occur under standard pricing and reimbursement pathways. The re-emergence of such traditional features has had a negative impact on the speed of patient access, as assessments introduce additional delays. Consequently, it is evident that MYMI agreements have started losing their original objective: to ensure fast patient access to innovative products while effectively managing the healthcare budget.

### 3.3. How Are MYMI Agreements Expected to Develop in the Future?

So far, MYMI agreements have not been explicitly revised to account for the complexity of oncology combinations that contain two or more innovative products. Although MYMI agreements could theoretically cover products’ use in combination indications, we observe in practice that patient access to the combination still requires the manufacturer of the other, non-PD-(L)1 component to negotiate a separate deal on top of the PD-(L)1, thus adding complexity and delays [17]. This delay is evident for oncology combination therapies in general: such therapies, on average, require 99 more days than other therapies between regulatory approval and the reimbursement decision across European countries [18]. New solutions may be needed in the future as the number of combination indications in oncology continues to increase.

The future relevance of MYMI for PD-(L)1s may, however, be impacted by payers’ anticipation of the loss of exclusivity of particular products. It is currently uncertain how the appetite for MYMI agreements will evolve as we move closer to the genericisation of PD-(L)1s.

Finally, a surprising feature of MYMI is that it has not yet been applied to other products that also have multiple indications, such as in immunology, where we observe that drugs are increasingly being developed and launched in multiple indications. For example, dupilumab, an immunosuppressant, is already marketed in five indications [19] and is in Phase III trials or filed for approval in a further five [20]. Although PD-(L)1s have pioneered the concept, very little about the structure and characteristics of the implemented MYMI agreements is unique to the class. Ensuring their relevance and benefit to other products in the future will require early conversations with policymakers and payers to align on the overlap in challenges and thus overlap in potential solutions.

### 3.4. What Would the Ideal MYMI Look Like?

Although each MYMI needs to be tailored to the country in question, drawing from the findings above, it is possible to consider how an ideal MYMI agreement could be structured. Ultimately, an optimal agreement would address the three primary objectives: reduce time to access, reduce administrative burden or at least eliminate delay, and improve budget impact predictability.

Based on the country experiences, an ideal MYMI agreement would result in immediate or close to immediate access to new indications, an approach to value assessment that does not delay access, provision of an indication-weighted average price, and horizon scanning. Immediate access—as demonstrated by the example in Belgium—eradicates delays in time-to-market, thereby allowing patients affected by the respective disease to access medication faster. If indication-specific value assessment is required, a retrospective value assessment supports speed of access and could potentially decrease the administrative burden. A weighted-average pricing approach, which would involve setting a single price based on the value of each indication, would help preserve financial benefits and incentives, avoiding indication-specific negotiations for both the payers and the manufacturers, as was discussed in the Swiss agreements. Finally, horizon-scanning implementation will help to improve budget predictability, as the payers can be prepared for any incoming changes to the market landscape.

An ideal system has several prerequisites: a process to negotiate and agree and renew the terms of the MYMI, good communication between companies and payers and the involvement of other stakeholders as appropriate, and a tracking system to understand the use of the indication. While the latter must be implemented with caution, as it may exacerbate the administrative burden, it also has some additional benefits beyond MYMI: tracking information will be helpful in the context of pricing and reimbursement of medicines used in combination and as monotherapy [21]. In addition, it also provides information about what treatment patients receive and whether it corresponds with the standard of care.

Finally, the main objective of finding new ways of pricing and reimbursement agreements for innovation is timely, affordable patient access. MYMI is one efficient solution, but one should not forget that there are other steps, beyond reimbursement, to achieve patient access [22].

## 4. Conclusions

The access challenges associated with oncology medicines have been exacerbated by ongoing innovation, such as the growing number of indications and availability of combination therapies, and by ongoing economic constraints that are impacting healthcare systems more broadly. Despite the fact that there has been no formal evaluation of the impact of MYMI agreements, conceptually, we consider the main benefits to be speed and breadth of access to treatment and budget predictability. Since 2017, as more evidence has become available regarding the potential benefits of MYMI agreements to patients, healthcare systems, and innovators, their adoption has expanded and evolved, and the launch of new indications has become smoother. For instance, from our perspective, the benefits of the Dutch MYMI agreement are the efficiencies created for the HTA body (ZIN), and thus the potential accelerated reimbursement of new indications. On the other hand, in Greece the key benefit seems to be budget predictability for the health system. We believe this suggests that MYMI agreements are viewed as successful, benefiting some or all involved stakeholders, such as patients and the healthcare system, and not viewed as a one-off. In other words, MYMI agreements appear to be here to stay, and experience has demonstrated that once a workable solution is found in a country, it can be sustained over many years and over multiple changes in the broader healthcare system and its associated pressures. However, it has also become apparent that MYMI agreements cannot be considered a one-size-fits-all solution, with evidence that countries are adapting the MYMI concept to suit the individual challenges and needs of each healthcare system. In addition, as experience shows, there is a risk that administrative steps take over and jeopardise the original impact of MYMIs. As PD-(L)1s continue to be developed and launched in an increasing number of indications and an increasing number of complex combinations, MYMI will remain a useful tool to enable patient access.
